# Haemorrhagic Shock After Iatrogenic Deep Circumflex Iliac Artery Injury During Paracentesis: A Rare Lethal Complication

**DOI:** 10.7759/cureus.59428

**Published:** 2024-04-30

**Authors:** Farah Marzuki, Guo Hou Loo, Nik Farhan Nik Fuad, Nik Ritza Kosai

**Affiliations:** 1 Upper Gastrointestinal and Metabolic Surgery Unit, Department of Surgery, Hospital Canselor Tuanku Muhriz, Universiti Kebangsaan Malaysia, Kuala Lumpur, MYS; 2 Interventional Radiology, Department of Radiology, Hospital Canselor Tuanku Muhriz, Universiti Kebangsaan Malaysia, Kuala Lumpur, MYS

**Keywords:** angioembolization, interventional radiology, coagulopathy, peritoneal tapping, ascites

## Abstract

Abdominal paracentesis is a commonly performed bedside procedure. It serves as a therapeutic and diagnostic tool for a variety of conditions. It is regarded as a safe procedure with a low risk of complications. Rarely, iatrogenic complications such as peritonitis, haemorrhage, and bowel perforation may occur. Intraperitoneal haemorrhage is rare and usually occurs due to bleeding from the intraabdominal venous collateral vessels or mesenteric varices. However, intraperitoneal haemorrhage secondary to injury to the abdominal wall arteries, such as the inferior epigastric artery or deep circumflex iliac artery (DCIA), is very uncommon.

We report on a 64-year-old man with decompensated cardiac failure who underwent paracentesis due to gross ascites. Twenty-four hours post-procedure, he became progressively hypotensive and lethargic. An ecchymosis measuring 3 cm × 2 cm was seen over the puncture site. An urgent CT angiography of the abdomen showed a large left-sided intraperitoneal haematoma with active contrast extravasation from the left DCIA. We performed a successful angioembolisation of the left DCIA.

It is important to note that intraperitoneal haemorrhages secondary to DCIA injury may present as occult intraperitoneal haemorrhage. Angioembolisation is a useful tool in the management of uncontrolled intraperitoneal haemorrhage. The recommended puncture site is in the left lower quadrant, 2-4 cm superior and medial to the anterior superior iliac spine (ASIS).

This case report serves to emphasise the rare but potentially lethal complication of a commonly performed procedure. A high index of suspicion of intraperitoneal haemorrhage is required for patients with unexplained hypotension post-paracentesis, even if overt abdominal signs are absent. The use of ultrasound guidance will aid in reducing the risk of severe complications and increasing the overall success rate.

## Introduction

Abdominal paracentesis is a commonly performed bedside procedure [1]. It serves as a therapeutic and diagnostic tool for a variety of conditions. It is regarded as a safe procedure with a low risk of complications, regardless of whether a physician or a trained nurse performs it [1,2]. Image-guided paracentesis is rarely required, and the safest site to perform this procedure is in the left lower quadrant of the abdomen [3-6]. Prior studies have shown that large-volume paracentesis is safe, with a risk of overall complications of approximately 1% [6,7].

Rarely, iatrogenic complications such as peritonitis, haemorrhage, and bowel perforation may occur, leading to significant patient morbidity and even mortality [8]. Intraperitoneal haemorrhage is rare and usually occurs due to bleeding from the intraabdominal venous collateral vessels or mesenteric varices [1,6]. This is a serious complication that needs urgent intervention [8]. There have been reported cases of intraperitoneal haemorrhage secondary to injury to the abdominal wall arteries, such as the inferior epigastric artery or deep circumflex iliac artery (DCIA) [8]. Keeping this in mind, we report a case of haemorrhagic shock caused by an iatrogenic injury to the DCIA following a therapeutic paracentesis.

## Case presentation

A 64-year-old man with dilated cardiomyopathy presented with symptoms of progressive decompensated cardiac failure. Clinically, he was tachypnoeic with an oxygen saturation of 90% on room air. Lung auscultation revealed crepitations until midzone in both lung fields, with bilateral lower limb pitting oedema. His abdomen was grossly distended with a positive fluid thrill. His initial blood gas test revealed type 1 respiratory failure, and his kidney function test showed an acute kidney injury. A diagnosis of decompensated cardiac failure was made, and he was started on oxygen supplementation, fluid restriction, and diuretics. He was nursed in the high-dependency ward, and as his abdomen was grossly distended and interfering with his respiratory efforts, a decision was made for a therapeutic paracentesis.

The procedure was carried out in the usual manner with an 18G-sized intravenous catheter (Vasofix®, B. Braun, Germany) under an aseptic technique. The catheter was inserted in the left lower quadrant of the abdomen, and it was a single puncture with no technical difficulties. We drained two litres of clear, straw-coloured ascitic fluid and removed the catheter post-procedure. There was no overt bleeding from the puncture site, and it was dressed with dry gauze. As this was a therapeutic paracentesis, the ascitic fluid was not sent for biochemical or microbiological analysis. Post-procedure, the patient remained well, less tachypnoeic, and there were no immediate complications. After 24 hours, he became progressively hypotensive and appeared more lethargic. The patient did not complain of any abdominal pain or worsening abdominal distension. An ecchymosis measuring 3 cm × 2 cm was seen over the puncture site, but there was no overt external bleeding. An urgent blood test revealed haemoglobin of 3.1 g/dL and a prolonged prothrombin time of 24.9 seconds with an international normalised ratio of 2.34. The baseline coagulation profile was within the normal range. A presumptive diagnosis of haemorrhagic shock secondary to intraperitoneal haemorrhage was made, and the patient was resuscitated with blood products. 

**Figure 1:**
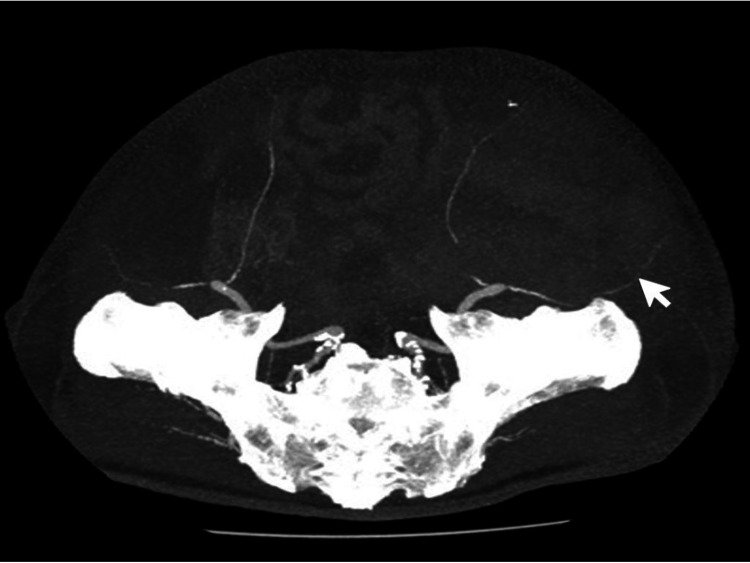
Computed tomography-angiographic (maximum-intensity projection) image of the arterial phase showing the course of the left deep circumflex iliac artery (white arrow) arising from the left external iliac artery.

**Figure 2:**
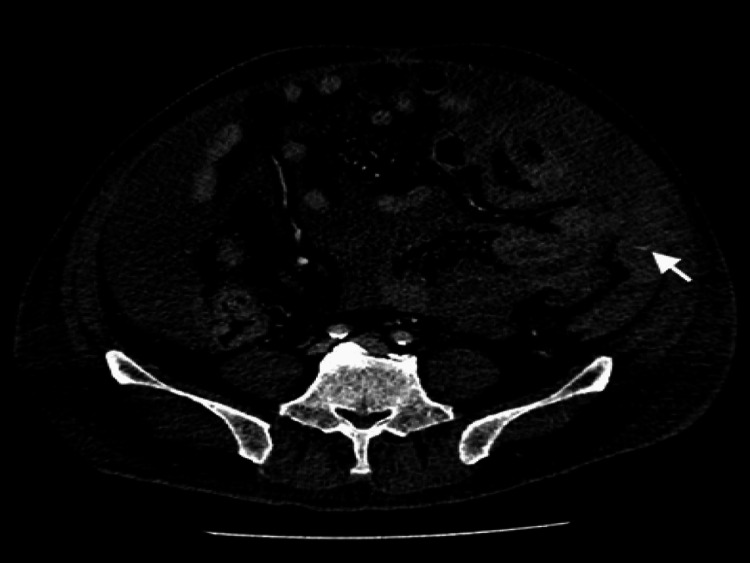
CT lower abdomen - pelvis (venous phase) image showing extravasation of contrast material (white arrow) within a hematoma in the left paracolic gutter, along the course of the deep circumflex iliac artery.

**Figure 3:**
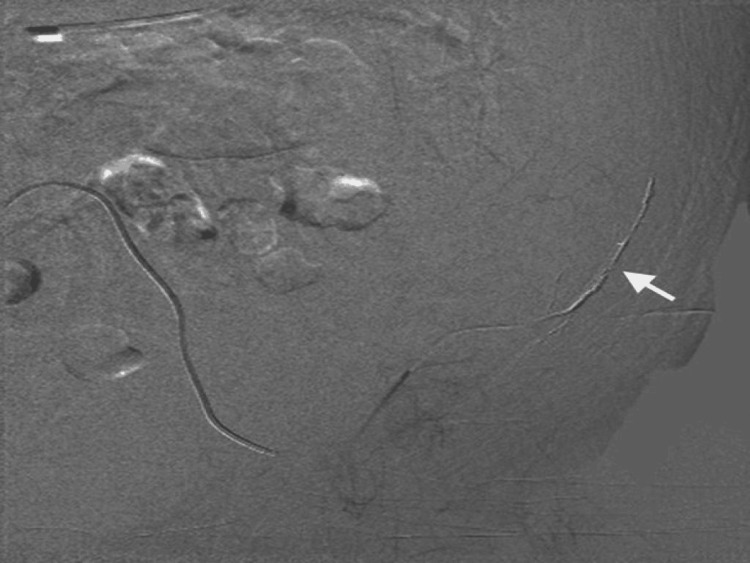
Successful angioembolisation of the left deep circumflex iliac artery (white arrow) performed using histoacryl/lipiodol mixture.

An urgent computed tomography (CT) angiography of the abdomen was performed, and it showed a large left-sided intraperitoneal haematoma with active contrast extravasation from the left DCIA (Figures 1-2). We performed an angioembolization of the left DCIA via the left femoral artery approach with histoacryl and lipiodol mixture, and post-procedure, no active contrast extravasation was seen (Figure 3). There was no further drop in haemoglobin subsequently. He was nursed in the intensive care unit post-procedure, but unfortunately, he deteriorated and developed multiorgan failure. He succumbed to a hospital-acquired infection after two weeks.

## Discussion

Paracentesis is a procedure in which a needle is inserted into the peritoneal fluid to draw out ascitic fluid, either for diagnostic or therapeutic purposes [9]. In refractory ascites, patients often require paracentesis to reduce abdominal discomfort and respiratory distress [9]. Although generally a safe procedure, contraindications to paracentesis include patients with disseminated intravascular coagulation and acute abdomens requiring surgery [9].

Iatrogenic injury due to paracentesis is a rare but potentially lethal complication [1]. It is reported that the complication rate from this procedure is 1%, and serious complications include haemorrhage, peritonitis, and bowel perforation [8]. The aetiology of post-paracentesis haemorrhage includes direct injury to the abdominal wall arteries, collateral veins, and rupture of mesenteric varices [1,8]. In a systematic review by Sharzehi et al., haemorrhagic complications were categorised into three groups: abdominal wall haematoma, intraperitoneal haemorrhage, and pseudoaneurysm [8]. Abdominal wall haematoma is the most common complication (52%), followed by intraperitoneal haemorrhage (41%), and pseudoaneurysm (7%) [8]. Injury to the inferior epigastric artery is the most frequent cause of haemorrhage, causing abdominal wall haematoma. This pattern of injury is more common in patients with ascites, as the inferior epigastric artery is displaced laterally due to the stretching of the abdomen [4].

In patients with ascites secondary to acute chronic liver disease who underwent paracentesis, severe coagulopathy and low fibrinogen levels are thought to increase the risk of severe haemorrhagic complications [10]. Intraperitoneal haemorrhage may be difficult to diagnose. It is commonly associated with rupture of the mesenteric varices or venous collaterals following large volume paracentesis, in which there is a shift in the pressure gradient of the venous circulation leading to dilation and subsequent rupture of these vessels [1]. The slow, continuous venous bleed gives little clinical signs, and the patient may present with shock [1]. The DCIA is one of the abdominal wall arteries arising from the external iliac artery or the femoral artery [4]. It is important to note that intraperitoneal haemorrhages secondary to DCIA injury may present as occult intraperitoneal haemorrhage rather than palpable abdominal wall haematoma [4]. 

Angioembolisation is a useful tool in the management of uncontrolled intraperitoneal haemorrhage, with less morbidity compared to open surgery [4]. It can also simultaneously detect the exact bleeding point, which may be difficult in open surgery [4]. Others have also reported successful angioembolization following life-threatening complications from injuries to the DCIA [8].

With knowledge of the origin of the DCIA and its course, this injury can be avoided. The recommended puncture site is in the left lower quadrant, 2-4 cm superior and medial to the ASIS [4]. The anterior superior iliac spine is easily palpated as the most prominent bony protuberance at the lateral end of the inguinal fold [11]. This puncture site, in relation to the ASIS, should be sufficient to avoid both the DCIA and the inferior epigastric artery. Ultrasound guidance, if available, should be used for paracentesis to reduce the risk of serious complications such as bleeding [5]. Ultrasound may also be used to assess the volume of intraperitoneal fluid and thereby increase the overall success rate of the procedure [5].

## Conclusions

This case serves to emphasise the rare but potentially lethal complication of a commonly performed procedure. A high index of suspicion of intraperitoneal haemorrhage is required for patients with unexplained hypotension post-paracentesis, even if overt abdominal signs are absent. Despite the low risk of occurrence, every clinician performing this procedure should be aware of this possible lethal complication. The use of ultrasound guidance will aid in reducing the risk of severe complications and increasing the overall success rate.
